# Predictors and correlates for weight changes in patients co-treated with olanzapine and weight mitigating agents; a post-hoc analysis

**DOI:** 10.1186/1471-244X-9-12

**Published:** 2009-03-28

**Authors:** Virginia L Stauffer, Ilya Lipkovich, Vicki Poole Hoffmann, Alexandra N Heinloth, H Scott McGregor, Bruce J Kinon

**Affiliations:** 1 Neuroscience, Lilly USA, LLC, Indianapolis, IN 46285, USA; 2 Statistics, Eli Lilly and Company, IN 46285, USA; 3 Neuroscience, Eli Lilly and Company, IN 46285, USA; 4 i3Statprobe, subsidiary of United Health Group, Ann Arbor, MI, USA

## Abstract

**Background:**

This study focuses on exploring the relationship between changes in appetite or eating behaviors and subsequent weight change for adult patients with schizophrenia or bipolar disorder treated with olanzapine and adjunctive potential weight mitigating pharmacotherapy. The aim is not to compare different weight mitigating agents, but to evaluate patients' characteristics and changes in their eating behaviors during treatment. Identification of patient subgroups with different degrees of susceptibility to the effect of weight mitigating agents during olanzapine treatment may aid clinicians in treatment decisions.

**Methods:**

Data were obtained from 3 randomized, double-blind, placebo-controlled, 16-week clinical trials. Included were 158 patients with schizophrenia or bipolar disorder and a body mass index (BMI) ≥ 25 kg/m^2 ^who had received olanzapine treatment in combination with nizatidine (n = 68), sibutramine (n = 42), or amantadine (n = 48). Individual patients were analyzed for categorical weight loss ≥ 2 kg and weight gain ≥ 1 kg. Variables that were evaluated as potential predictors of weight outcomes included baseline patient characteristics, factors of the Eating Inventory, individual items of the Eating Behavior Assessment, and the Visual Analog Scale.

**Results:**

Predictors/correlates of weight loss ≥ 2 kg included: high baseline BMI, low baseline interest in food, and a decrease from baseline to endpoint in appetite, hunger, or cravings for carbohydrates. Reduced cognitive restraint, increase in hunger, and increased overeating were associated with a higher probability of weight gain ≥ 1 kg.

**Conclusion:**

The association between weight gain and lack of cognitive restraint in the presence of increased appetite suggests potential benefit of psychoeducational counseling in conjunction with adjunctive pharmacotherapeutic agents in limiting weight gain during antipsychotic drug therapy.

**Trial Registration:**

This analysis was not a clinical trial and did not involve any medical intervention.

## Background

In adult patients with serious and persistent mental illnesses such as bipolar disorder or schizophrenia, obesity is a common comorbidity. [1] Many antipsychotic medications used to treat these diseases are associated with an increased risk of weight gain. A meta-analysis by Allison and colleagues showed a significantly greater incidence of weight gain in patients treated with clozapine or olanzapine compared with patients treated with other atypical antipsychotics. [2] Since 1996, the United States (US) prescribing information for olanzapine has advised clinicians of the potential for significant weight gain in more than 1/4 of patients during short-term therapy and in more than 1/2 of patients who receive long-term olanzapine therapy. The current prescribing information for olanzapine warns clinicians of the potential for short- and long-term weight gain during treatment. [3] Treatment-emergent weight gain may influence both the physical health of the patient and treatment continuation. Considering the high obesity rates in the US general population (32.9%) [4] and in patients with schizophrenia (42%), [5] the potential risk of weight gain needs to be evaluated carefully.

Recently, the Clinical Antipsychotic Trials of Intervention Effectiveness (CATIE) study evaluated the overall treatment effectiveness of olanzapine, perphenazine, quetiapine, risperidone, and ziprasidone. In this study, patients treated with olanzapine showed the greatest treatment effectiveness as determined by measuring the length of time patients remained on their prescribed medication. Patients treated with olanzapine remained on their medication statistically significantly longer compared to patients treated with quetiapine or risperidone, but not compared to patients treated with perphenazine or ziprasidone. [6] However, olanzapine-treated patients gained significantly more weight than patients in the other treatment groups (p < .001), and significantly more patients treated with olanzapine reported potentially clinically significant weight gain ≥ 7% increase from baseline weight (p < .001) and discontinued treatment due to weight gain or changes in metabolic parameters (p < .001). [6]

In light of these data, clinicians are searching for effective strategies to help manage potential weight gain in this patient population. While one option is to switch to another antipsychotic medication that may have a more favorable weight gain profile, this does not always reverse the weight gain the patient may have already experienced. [7] Behavioral therapy and pharmacologic treatments have been studied as alternatives to switching antipsychotic medications in order to potentially limit or reverse weight gain during treatment with olanzapine. Recently, Ganguli published a comprehensive review summarizing behavioral therapy to induce weight loss in patients with schizophrenia. [8] This review showed that non-pharmacologic interventions were successful in controlling weight in some patients, and it concluded that all weight maintenance efforts should include behavioral interventions, dietary advice, and exercise. In addition, the Cochrane Group recently conducted a comprehensive review critically evaluating both non-pharmacologic and pharmacologic randomized controlled trials (RCTs) of adjunctive agents hypothesized to prevent weight gain or to reduce weight in patients with schizophrenia who were receiving antipsychotic treatment. Within the group of RCTs that were included in this review, studies using cognitive/behavioral therapy showed the best efficacy in weight prevention (weighted mean difference [WMD]: -3.38 kg) and, to a lesser extent, in weight reduction (WMD: -1.69 kg). Pharmacological intervention studies resulted in a more modest prevention of weight gain (WMD: -1.16 kg). They concluded that modest weight loss can be achieved in patients with schizophrenia by pharmacological and non-pharmacological interventions, but this conclusion is limited by the small number of studies available and the substantial heterogeneity across studies. [9]

A comprehensive review of weight mitigating agents and their use during treatment with antipsychotics has been published recently by Baptista and colleagues. [10]

This study focuses on pharmacological interventions and their ability to prevent weight gain or to induce weight loss when combined with olanzapine treatment. The aim is neither to extract predictors for weight change during olanzapine monotherapy nor to compare different weight mitigating agents, but to evaluate patients' characteristics and changes in their eating behaviors during treatment with olanzapine and weight mitigating agents in overweight patients. These predictors may be useful in identifying subgroups of patients who may be susceptible to the effect of weight mitigating agents during olanzapine treatment.

Previous studies of the effect of weight mitigating agents focused on evaluating treatment difference in weight changes, which were often statistically non-significant [11,12] and might explain the modest effects seen in the analysis conducted by the Cochrane Group. [9] In contrast, we defined categorical outcomes that constitute clinically significant weight loss and weight gain during treatment. In our opinion, these categorical analyses provide information that is clinically more useful than analyses based on mean weight changes. We hypothesized that, in patients who received weight-mitigating agents during olanzapine treatment, the presence or absence of cognitive restraint and changes in eating behaviors may both be indicators of subsequent weight loss or weight gain. To evaluate this hypothesis, and to also identify any relevant demographic characteristics predictive of the outcome, we performed post-hoc exploratory analyses of patients who received olanzapine treatment in combination with 1 of 3 weight-mitigating agents (nizatidine, sibutramine, or amantadine) in 3 Eli Lilly and Company-sponsored, placebo-controlled, weight-mitigation studies. These studies were selected because the complete datasets allowed the examination of potential predictors of weight change and, therefore, could help identify patients who might or might not be more susceptible to weight change when receiving a pharmacologic treatment. We evaluated the association between appetite, eating behaviors (both at baseline and post-treatment), and weight change in patients with schizophrenia or bipolar disorder treated with olanzapine and an adjunctive pharmacotherapy for the purpose of identifying potential predictors and correlates for weight changes.

## Methods

The analyses presented here utilize data from 3 clinical trials sponsored by Eli Lilly and Company, in adult patients with a Diagnostic and Statistical Manual of Mental Disorders, Fourth Edition – Text Revision (DSM-IV-TR) diagnosis of schizophrenia, schizoaffective disorder, schizophreniform disorder, or bipolar disorder, that examined the effects of nizatidine, sibutramine, or amantadine compared to placebo on weight change. Only data from those 3 trials were included due to the fact that the authors could not access additional datasets to the extent necessary. The primary results from each study have been previously published in peer-reviewed journals [13,14] or as a clinical trial registry (CTR) summary (, Trial ID: 5102). All study protocols were reviewed and approved by the appropriate Institutional Review Boards at each study site before enrolling any patient. Conduct of the studies was in accordance with the Declaration of Helsinki, the US Federal Drug Administration Code of Federal Regulations (21 CFR, Part 50), and Good Clinical Practices. All eligible participants provided written informed consent before undergoing any study procedure or receiving any study treatment.

### Patients

From the pool of participants who were enrolled in these 3 clinical studies, patients with a baseline BMI ≥ 25 kg/m^2 ^who were receiving treatment with olanzapine and were randomized to 1 of the adjunctive weight-mitigating agents or placebo were included in these analyses. In addition, the 16-week time point was used as a common endpoint. Detailed study design information, including inclusion and exclusion criteria, can be found in the primary publications for the nizatidine and amantadine studies [13,14] and in the CTR summary for the sibutramine study (, Trial ID: 5102). The sibutramine and the amantadine studies were designed as weight-reduction studies (i.e., patients had already experienced a pre-specified threshold of weight gain while receiving olanzapine treatment), while the nizatidine study evaluated weight gain prevention after initiation of olanzapine treatment.

#### Nizatidine study

In this double-blind, placebo-controlled trial, 175 male and female patients, 18–65 years of age, were randomly allocated in a 1:1:1 ratio to receive either open-label olanzapine (5–20 mg/day, flexible dosing) combined with double-blinded nizatidine (150 mg/day or 300 mg/day) or placebo for 16 weeks. All patients had been diagnosed with schizophrenia, schizoaffective disorder, or schizophreniform disorder.

#### Sibutramine study

In this double-blind, placebo-controlled study, 83 male and female patients, 18–65 years of age, were randomly allocated to receive either open-label olanzapine (5–20 mg/day, flexible dosing) combined with double-blinded sibutramine (3 weeks 10 mg/day, fixed dose; 3 weeks dose adjustment 5–15 mg/day, flexible dose; 10 weeks 5–15 mg/day, fixed dose) or placebo over 16 weeks. Due to enrollment difficulties, the study was terminated before the original enrollment goal of 170 patients had been met. All patients had been diagnosed with schizophrenia, schizoaffective disorder, schizophreniform disorder, or bipolar I disorder.

#### Amantadine study

In this double-blind, placebo-controlled trial, 125 male and female patients ages 18–65 years, were randomly assigned to receive either open-label olanzapine (5–20 mg/day, flexible dosing) combined with double-blinded amantadine (100–300 mg/day, flexible dosing) or placebo. At the end of the 16-week study period, an 8-week double-blind extension period followed during which patients continued to receive open-label olanzapine and double-blind adjunctive treatment with amantadine. All patients met the diagnostic criteria for schizophrenia, schizoaffective or schizophreniform disorder, or bipolar I disorder.

### Definition of Outcomes

For the purpose of these analyses, we defined a priori successful outcome as the occurrence of ≥ 2 kg weight loss and unsuccessful outcome as ≥ 1 kg weight gain. We discriminated between weight loss and weight gain at any time during the study versus weight loss and weight gain sustained to the 16-week endpoint or to study discontinuation.

### Eating Behavior Assessment

Outcome measures included 3 eating assessment scales: the Eating Inventory (EI), [15] the Eating Behavior Assessment (EBA, a Lilly-developed scale, not validated), and the Visual Analog Scale (VAS). Since the focus of our analyses was on predictors and correlates rather than treatment comparisons, clinically meaningful, non-validated scales are acceptable explanatory variables for use in a Cox proportional hazards regression. The EI is a 51-item questionnaire that measures 3 factors: cognitive restraint of eating, disinhibition of eating, and susceptibility to hunger. The EBA consists of 9 items and is used to determine eating behavior during the previous week, rated from 0 ("not at all," meaning the patient reported not experiencing the behavior/feeling at all) to 4 ("extremely," meaning the patient reported exceedingly experiencing the behavior/feeling). The VAS is used to determine eating behavior during the previous 24 hours and consists of 3 items (hunger, interest in food, and appetite) rated from 0 ("not at all," meaning the patient reported not experiencing the behavior/feeling at all) to 10 ("extremely," meaning the patient reported exceedingly experiencing the behavior/feeling). Weight, VAS score, and the EBA were measured at baseline and at Weeks 1–6, 8, 12, and 16. The EI was assessed at baseline and at Weeks 4, 8, 12, and 16.

### Statistical Analysis

For each study individually, the overall time to weight loss or weight gain was evaluated with a Kaplan-Meier product-limit estimator. To examine associations between measures of craving, eating factors, and eating behaviors and subsequent or concurrent weight loss or weight gain, a proportional hazards Cox regression with study-specific baseline hazard functions and time-varying covariates was employed, with disease (psychiatric diagnosis) as one of the baseline covariates in the model.

Important predictors and correlates were identified using stepwise variable selection in a Cox proportional hazards regression model. The original set of variables included changes from baseline and baseline values for eating scales, BMI, ethnicity, gender, and age. Only the results for the final models selected are reported. No subgroup analyses were performed discriminating between patients with schizophrenia and those with bipolar disorder, as the resulting sample sizes would be too small to produce meaningful results. All statistical analyses are reported with a significance level of p < .05.

## Results

### Patients

A total of 158 patients met the a priori selection criteria for the analyses presented here. Table 1 summarizes the patient characteristics at baseline. The nizatidine study provided the highest number of patients (n = 68), followed by the amantadine (n = 48) and the sibutramine (n = 42) studies.

**Table 1:** Patient Characteristics at Baseline

	**Nizatidine** **Study** **(n = 68)**	**Sibutramine Study** **(n = 42)**	**Amantadine Study** **(n = 48)**	**Total** **(N = 158)**
	
Age, years; mean (SD)	43.5 (10.2)	38.7 (11.6)	40.6 (12.0)	41.3 (11.3)
Weight, kg; mean (SD)	85.1 (12.2)	99.8 (19.7)	95.1 (18.9)	92.1 (17.7)
BMI, kg/m^2^; mean (SD)	30.1 (3.8)	35.0 (5.7)	32.3 (5.4)	32.1 (5.2)
Age at disease onset, years; mean (SD)	25.5 (7.5)	23.6 (9.8)	24.6 (10.6)	24.7 (9.1)
BPRS; mean (SD)	21.0 (14.1)^a^	8.3 (6.8)	11.6 (8.3)	14.6 (12.1)
Caucasian race, %	67.6	78.6	81.3	74.7
Gender, % males	60.3	35.7	47.9	50.0
EBA Total; mean (SD)	11.4 (4.8)^b^	19.2 (6.2)	19.7 (7.9)	16.0 (7.4)
VAS (hunger); mean (SD)	4.2 (2.2)^c^	5.7 (2.3)	6.1 (2.3)	5.2 (2.4)
VAS (interest in food); mean (SD)	4.8 (2.7)^c^	5.4 (2.5)	6.4 (2.9)	5.4 (2.8)
VAS (appetite); mean (SD)	4.9 (2.4)^c^	5.9 (2.6)	6.7 (2.6)	5.7 (2.6)
Eating Inventory (cognitive restraint); mean (SD)	7.1 (4.1)^b^	8.1 (3.1)	7.4 (3.9)	7.5 (3.8)
Eating Inventory (disinhibition); mean (SD)	5.0 (3.0)^b^	8.2 (3.8)	7.4 (3.3)	6.6 (3.6)
Eating Inventory (hunger); mean (SD)	5.2 (2.9)^b^	8.4 (3.1)	8.3 (3.3)	7.0 (3.4)

Abbreviations: BMI = body mass index; BPRS = Brief Psychiatric Rating Scale; EBA = Eating Behaviors Assessment: total of 9 items; each item is rated from 0 (not at all) to 4 (extremely); EI = Eating Inventory; SD = standard deviation; VAS = Visual Analog Scale: measurements are points on a scale from 0 (not at all) to 10 (extremely).

^a^n = 65

^b^n = 67

^c^n = 66

### Eating Inventory and EBA

To better understand the relationships among the different measures of eating behaviors and attitudes, we computed pairwise correlations between the 3 factors of the EI as well as correlations between these factors and the items from EBA. At baseline, the 3 factors of the EI had the following correlations within the pooled data (N = 157; 1 patient had missing data at baseline): r = .222 (p = .0051) between factor I (Cognitive Restraint) and factor II (Disinhibition); r = .0025 (p = .9753) between factor I and factor III (Hunger); and r = .675 (p < .0001) between factor II and factor III. Note that if factor I and factor II measured opposite items as might be assumed in a model in which patients with more cognitive restraint have less disinhibition, one would expect a negative correlation between these factors, whereas our results showed a mild positive correlation (see Discussion pp.17–18).

Pearson correlations between the 3 factors of the EI with items from the EBA at baseline are shown in Table 2. Highly significant correlations were observed for all items from EBA with factor III (Hunger) and significant to highly significant correlations for most items from EBA with factor II (Disinhibition).

**Table 2:** Correlations of EI Factors with Items from EBA

**Eating Behavior Assessment Items**	**FACTOR I** **"Cognitive Restraint"**	**FACTOR II** **"Disinhibition"**	**FACTOR III** **"Hunger"**
1. How hungry have you been?	5.5	17.3^a^	28.5^c^
2. How strong has your appetite been?	3.8	17.2^a^	38.7^c^
3. Have you craved sweets or other carbohydrates?	2.2	29.2^c^	40.4^c^
4. Have you craved fatty foods?	5.6	15.9^a^	23.2^b^
5. When you finished a meal, have you felt full or satisfied?	15.3	-2.5	-21.1^b^
6. Does it take an excessive amount of food before you feel satisfied?	5.7	23.3^b^	35.3^c^
7. Have you been thinking about food?	15.4	41.9^c^	41.6^c^
8. Have you been overeating?	0.3	39.0^c^	43.9^c^
9. Do you feel your eating is out of control?	-11.2	44.3^c^	43.6^c^
Total score	6.8	41.5^c^	49.2^c^

Abbreviations: EBA = Eating Behaviors Assessment; EI = Eating Inventory.

Values in %

Pearson correlations × 100 adjusted for study effect and baseline BMI

n = 150

^a^p < .05

^b^p < .01

^c^p < .001

### Weight Outcomes

**Figure 1:**
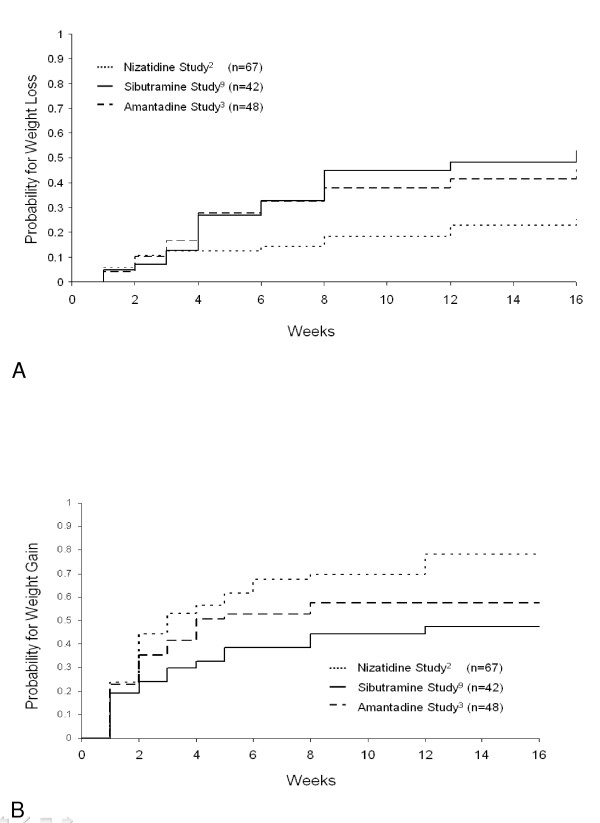
**Time to weight loss/gain**. 1a) Kaplan-Meier estimates of cumulative probability for weight loss ≥ 2 kg, by study. 1b) Kaplan-Meier estimates of cumulative probability for weight gain ≥ 1 kg, by study.

Analysis of weight outcomes within the individual studies revealed that the highest percentage of patients who experienced successful weight loss at any time (42.9%; 18/42) was in the sibutramine study, while the highest percentage of patients who showed successful weight loss sustained to endpoint (33.3%; 16/48) was in the amantadine study. The highest percentages of weight gain were observed in the nizatidine study, with 70.2% (47/67) of patients showing weight gain at any time and 59.7% (40/67) whose weight gain was sustained to endpoint (Table 3). Figure 1 illustrates the time to weight loss (Figure 1a) and to weight gain (Figure 1b) in the individual study populations. While Figure 1 summarizes the results, it is not intended to suggest direct comparisons of the efficacies of the different weight mitigating agents used in our analyses.

**Table 3:** Summary of Weight Outcomes

	**Weight Loss ≥ 2 kg**		**Weight gain ≥ 1 kg**	
	
	**At any time**	**Sustained to endpoint**	**At any time**	**Sustained to endpoint**
	
Nizatidine study (n = 67)	20.9% (n = 14)	7.5% (n = 5)	70.2% (n = 47)	59.7% (n = 40)
Sibutramine study(n = 42)	42.9% (n = 18)	26.2% (n = 11)	42.9% (n = 18)	19.1% (n = 8)
Amantadine study(n = 48)	39.6% (n = 19)	33.3% (n = 16)	56.3% (n = 27)	37.5% (n = 18)

Only patients with at least 1 post-baseline measurement are included.

### Significant Weight Predictors

We were able to identify five significant predictors for weight loss in patients treated with olanzapine and 1 of the 3 weight-mitigating agents; 2 of these were baseline variables while 3 were time-dependent variables: higher baseline BMI, less interest in food at baseline, decrease in appetite, decrease in cravings for carbohydrates, and decrease in hunger (Table 4). On the other hand, 3 time-dependent variables were significantly correlated with weight gain in our patient cohort: decrease in cognitive restraint, increase in hunger, and increase in overeating (Table 5).

**Table 4:** Significant Predictors of Weight Loss

**Variable**	**HR**	**95% CI**	**Interpretation**
Baseline BMI	1.09^b^	1.03–1.15	Patients with higher baseline BMI were more likely to lose weight, and patients with lower baseline BMI were less likely to lose weight
Baseline VAS2 (interest in food)	0.81^c^	0.73–0.91	Patients who had less interest in food were more likely to lose weight and patients who had more interest in food were less likely to lose weight
Change from baseline in appetite (EBA-2)	0.65^b^	0.48–0.86	Patients experiencing a decrease in appetite were more likely to lose weight and patients experiencing an increase in appetite were less likely to lose weight
Change from baseline in cravings for sweets or other carbohydrates (EBA-3)	0.75^a^	0.59–0.94	Patients experiencing decreased craving for carbohydrates were more likely to lose weight and patients experiencing increased craving for carbohydrates were less likely to lose weight
Change from baseline in hunger (VAS1)	0.87^a^	0.76–0.99	Patients experiencing a decrease in hunger were more likely to lose weight and patients experiencing an increase in hunger were less likely to lose weight

Abbreviations: BMI = body mass index; CI = confidence interval; EBA = Eating Behaviors Assessment; HR = hazard ratio; VAS = Visual Analog Scale.

Pooled analysis across all 3 studies

HR was adjusted for study effect and other variables included in the model

^a^p < .05, ^b^p < .01, ^c^p < .001

**Table 5:** Significant Predictors of Weight Gain

**Variable**	**HR**	**95% CI**	**Interpretation**
Change from baseline in FACTOR 1 (Cognitive Restraint)	0.81^b^	0.73–0.90	Patients experiencing a decrease in cognitive restraint were more likely to gain weight and patients experiencing an increase in cognitive restraint were less likely to gain weight.
Change from baseline in EBA-1 (Hunger)	1.32^a^	1.07–1.64	Patients experiencing an increase in hunger were more likely to gain weight and patients experiencing a decrease in hunger were less likely to gain weight.
Change from baseline in EBA-8 (Overeating)	1.28^a^	1.07–1.53	Patients experiencing an increase in overeating were more likely to gain weight and patients experiencing a decrease in overeating were less likely to gain weight.

Abbreviations: CI = confidence interval; EBA = Eating Behaviors Assessment; HR = hazard ratio.

Pooled analysis across all 3 studies

HR was adjusted for study effect and other variables included in the model (baseline BMI was also included, although it was not significant, p = .543)

^a^p < .01;^b^p < .0001

## Discussion

In these post-hoc analyses, we examined the association between appetite, eating behavior, and weight change for patients with schizophrenia or bipolar disorder treated with olanzapine and one of three potential weight mitigating agents: nizatidine, sibutramine, or amantadine. We were able to extract predictors for weight loss and for weight gain in these patients. Additionally, we analyzed categorical weight loss and weight gain at any time during the study, and weight change maintained to endpoint for the individual study groups. These results varied widely among the 3 compounds studied. The analyses presented here did not focus on the phenomenon of weight gain as a treatment emergent adverse event during treatment with olanzapine, which has already been very well characterized. [2,3,6]

### Predictors for Weight Loss and Weight Gain

Five variables were identified as predictors for weight loss in patients treated with olanzapine and weight mitigating agents, 2 of which were baseline variables that existed in patients treated with olanzapine before the introduction of any weight-mitigating agent. These baseline predictors were: higher BMI and less interest in food. The other 3 predictors for weight loss were time-dependent variables that gained significance after initiation of treatment with weight-mitigating agents. Patients who experienced decreases in appetite, cravings for carbohydrates, or hunger were more likely to lose weight.

Interestingly, all significant predictors for weight gain in patients treated with olanzapine and weight mitigating agents were time-dependent variables, measured concurrently with weight gain after initiation of treatment. Patients who experienced a decrease in cognitive restraint and/or an increase in hunger and/or an increase in overeating were more likely to gain weight. The fact that none of the variables available prior to treatment (e.g. patient characteristics) were significant predictors of weight gain, suggests that identifying such patients prior to treatment with weight-mitigating agents may be a challenging task, and underscores the importance of regular patient monitoring during treatment.

To test the robustness of our predictor analysis, we repeated the analysis using a subset of the original patient population, excluding all patients from the nizatidine group. We chose to exclude the nizatidine group for this test, as it is the only weight gain prevention study among the 3 studies and therefore the trial with the most potential for bias. The results confirmed BMI and interest in food as baseline predictors for weight loss, as well as decreases in cravings for carbohydrates and/or hunger as time-dependent predictors for weight loss. Similarly, a decrease in cognitive restraint and an increase in overeating were identified as predictors for weight gain. This subgroup analysis did not yield the previously identified decrease in appetite as a predictor for weight loss and increase in hunger as a predictor for weight gain. Those factors seem to be of importance for potential weight changes in patients that have not yet experienced weight gain, as is the case with the nizatidine group. It appears that in patients that have already gained weight, as is the case for patients in the amantadine and sibutramine groups, these factors were not predictive for potential weight loss or weight gain in response to the addition of a weight mitigating agent. One explanation might be that changes in appetite and hunger play a more important role as predictors/correlates of changes in weight for patients at earlier stages of weight gain. Once patients have gained substantial weight, further fluctuations in appetite or hunger do not predict weight changes, but cognitive restraint and actual eating behavior seem to remain important predictive factors. However, more comprehensive analyses of larger populations of patients are needed to validate this hypothesis.

Our finding of a decrease in cognitive restraint (the cognitive control of eating) as a significant predictor for weight gain is especially interesting considering the findings of Khazaal and colleagues, who showed that patients with schizophrenia present with cognitive distortions (in their thinking about weight regulation and self control) regarding weight gain when compared with control individuals. [16] Additionally, they also demonstrated that cognitive behavioral therapy improved binge eating symptomatology and weight-related cognitive thinking in patients who had gained weight during treatment with antipsychotic drugs. These patients experienced more progressive weight loss after cognitive behavioral therapy than control patients. [17] To address and utilize the importance of cognitive restraint as a predictive factor, clinically relevant measures to monitor cognitive restraint in a given patient need to be developed.

Recently, Lipkovich and colleagues [18] reported early predictors of substantial weight gain in patients who were treated with olanzapine. Their analysis revealed that patients with bipolar disorder treated with olanzapine who had gained 2 to 3 kg in the first 3 weeks after initiation of treatment, were at higher risk for substantial weight gain after 30 weeks of treatment. These results are in agreement with another study that showed patients with early weight gain while receiving olanzapine treatment for schizophrenia, schizophreniform, or schizoaffective disorder (increases of at least 2 kg in the first 3 weeks of treatment) were more likely to develop substantial weight gain (>10 kg after 30 weeks) over the course of treatment. [19] Additionally, patients who did not experience this amount of early weight gain, but had a BMI ≥ 27 kg/m^2 ^at treatment initiation, were also at higher risk for substantial weight gain (>10 kg) after 30 weeks of treatment. [18] Similarly, lower BMI at baseline was identified as a predictor for weight gain during treatment with antipsychotics in additional studies. [20,21] Identification of the discussed risk factors may help clinicians to better focus weight management efforts on susceptible patients.

### Eating Behavior Assessment

In the analyses presented here, we utilized the 3-factor EI developed by Stunkard and Messick. [15] Their original work utilized 2 main cohorts, "dieters" and "free eaters", to validate and optimize the questionnaire and factor analysis. Comparison of our results with theirs revealed that correlations between eating factors evaluated in our patient sample were very similar to those in their cohort of "free eaters" (factor I to factor II: r = .222 versus r = .19; factor I to factor III: r = .0025 versus r = -.06; factor II to factor III: r = .675 versus r = .73). The majority of our patients are probably "free eaters", meaning that they do not routinely restrict their dietary intake in order to control their body weight.

Analysis of correlations between the 3-factor EI and the EBA yielded interesting results. While EI factor II (Disinhibition) and factor III (Hunger) showed highly significant correlations to EBA items, EI factor I (Cognitive Restraint) was only mildly correlated to 2 EBA items ("When you finished a meal, have you felt full or satisfied?" and "Have you been thinking about food?"). It appears that the dimension captured in factor I (Cognitive Restraint) of the EI is poorly represented on the EBA scale. At least in the patient population analyzed in the current study, factor I (Cognitive Restraint) forms a complementary dimension, it is not the opposite of factor II (Disinhibition), as the labeling of those dimensions might suggest. The EI and the EBA scales appear to complement one another in the evaluation of different dimensions of eating behavior. While it is a limitation of the current analyses that both EBA and VAS are non-validated scales, their usage within an exploratory analysis as presented here is appropriate as we did not attempt to examine treatment differences with those scales. Instead, they were used in conjunction with EI, a validated scale, to examine correlations between patient characteristics and susceptibility to weight change during treatment with olanzapine and weight mitigating agents.

### Categorical Weight Gain and Weight Loss

In this analysis, we chose the occurrence of ≥ 2 kg weight loss as an indication of a successful outcome and the occurrence of ≥ 1 kg weight gain as a sign for an unsuccessful outcome. While those cutoff points were defined without prior evidence for their validity, we believe that this categorical evaluation of the study populations allows valuable insights. No established cutoff criteria were available to serve as an alternative for our arbitrary cutoff points.

Analysis of categorical weight gain and weight loss within each of the individual study populations revealed that patients receiving adjunctive treatment with nizatidine showed the poorest weight control performance. This study had the lowest percentage of patients who experienced successful weight loss and the highest percentage of patients who gained weight throughout the course of the study. The patients treated with nizatidine had a slightly lower baseline BMI in comparison to the other 2 study populations, which might be an explanation for the lower proportion of patients who experienced weight loss. Also of note, the inclusion criteria varied between the 3 studies. Both the amantadine and sibutramine studies required an initial 5% to 7% weight gain while receiving olanzapine treatment prior to study entry, but the nizatidine study did not. The principal objective of the nizatidine study was weight prevention when initiating olanzapine treatment. It has been reported that patients initiated on olanzapine may experience most of their weight gain during the first 6 to 9 months of treatment. [22] Therefore, the previously mentioned inclusion criterion in the amantadine and sibutramine studies selected for patients that had potentially already experienced initial weight gain, while the patient population enrolled in the nizatidine study included patients who were just initiated on olanzapine treatment. Finally, we cannot exclude the possibility that nizatidine is less effective than amantadine or sibutramine as a weight-mitigating agent. Since the analysis presented here does not represent a direct statistical comparison of all 3 adjunctive treatments, further studies are needed. However, our result is in agreement with reports in the literature that did not show strong weight loss properties of nizatidine in double-blind, placebo-controlled studies. [11,23]

### Intervention Strategies

A limitation of the analyses presented here is that only patients with a BMI of ≥ 25 kg/m^2 ^were included, therefore the results cannot be generalized to patients with a BMI of <25 kg/m^2^. Additionally, the enrollment criteria for the studies we utilized did not take into account the individual patient's stage of treatment with olanzapine. While patients initiating olanzapine treatment may need to focus on weight gain prevention, those patients who have already experienced weight gain during olanzapine treatment may need to focus on weight reduction.

Generally, early intervention with the goal of weight gain prevention seems to be the more promising approach. Several studies suggest that lifestyle modifications resulting in reduced caloric intake and enhanced physical activity are helpful in minimizing weight gain during treatment with olanzapine in patients susceptible to weight gain, [8] while these measures are not effective in all patients. [24,25] Additionally, there are promising reports of successful prevention of weight gain with weight-mitigating agents in some patients. [14,26-29] Recently, Wu and colleagues demonstrated the efficacy of metformin in preventing weight gain temporally associated with olanzapine treatment in a randomized, placebo-controlled trial in drug-naïve, first-episode patients with schizophrenia. [30] However, for many patients, weight gain has already occurred and weight reduction is needed. This goal is more difficult to achieve. Some promising results point to the effectiveness of behavioral therapy and adjunctive pharmacotherapy in helping to achieve weight reduction. [8,23,31-40] In a randomized, placebo-controlled trial, metformin plus lifestyle intervention showed the best effect on weight loss. [41] This is some of the best empirical evidence to date for the efficacy of treating weight gain during treatment with antipsychotics. Overall, both weight gain prevention and weight reduction are important therapeutic goals in patients receiving olanzapine treatment.

To the best of our knowledge, this is the first analysis examining predictors for weight loss and weight gain in patients treated with olanzapine and weight-mitigating agents. Interestingly, baseline BMI has been identified in the past as a predictor for weight gain with olanzapine treatment, with patients with lower BMI tending to gain more weight during treatment with olanzapine. [21,42] The results of our analysis indicate that weight-mitigating agents (in particular nizatidine, amantadine and sibutramine) as adjunctive treatment to olanzapine therapy do not appear to be beneficial for all patients, but might have therapeutic potential for some patients. Prospective studies are needed to better identify patients who will benefit from such treatments. In addition, we suggest that this analytic approach may be beneficial as a secondary or post-hoc analysis of ongoing studies with potential pharmacologic agents to gain a better understanding of patients who may or may not respond to a particular strategy.

Clinicians can help mitigate the potential weight gain temporally associated with olanzapine treatment by being aware of the patient's characteristics like baseline BMI and baseline level of interest in food, and monitoring the patient early in treatment for weight gain, changes in appetite or hunger, or cravings for carbohydrates, and for reduced cognitive restraint. These factors can help the clinician determine when to intervene.

## Conclusion

In the present analyses we observed an association between weight gain and reduced cognitive restraint combined with increased appetite and overeating in some patients treated with olanzapine and an adjunctive weight-mitigating agent. This suggests that the combined approach of psychoeducational counseling aimed at behavior modification and pharmacologic weight-mitigating agents, for select patients, may be the most beneficial to limit weight gain during treatment with olanzapine. Further research in this area is warranted.

## Abbreviations

BMI: body mass index; CATIE: Clinical Antipsychotic Trials of Intervention Effectiveness; CFR: Code of Federal Regulations; CTR: clinical trial registry; DSM-IV-TR: Diagnostic and Statistical Manual of Mental Disorders, Fourth Edition-Text Revision; EBA: Eating Behavior Assessment; EI: Eating Inventory; FDA: Food and Drug Administration; kg: kilogram; RCTs: randomized controlled trials; VAS: Visual Analog Scale; WMD: weighted mean difference.

## Competing interests

This work was sponsored by Lilly USA, LLC, the manufacturer of olanzapine. Drs. Stauffer and McGregor are employees of Lilly USA, LLC. Drs. Lipkovich, Poole Hoffmann, and Kinon are employees of Eli Lilly and Company. Dr. Heinloth is a scientific writer employed full-time by i3 Statprobe, a division of Ingenix, which is a subsidiary of UnitedHealth Group.

## Authors' contributions

Authors VLS, VPH, HSM, and BJK conceived of the study, participated in its design and coordination, and were involved in the interpretation of the data. Author IL participated in the design of the study, performed the statistical analysis, and contributed to the interpretation of the data. ANH drafted the manuscript and contributed to the interpretation of the data. All authors read and approved the final manuscript.

## Pre-publication history

The pre-publication history for this paper can be accessed here:


